# Fatigue independently predicts different work disability dimensions in etanercept-treated rheumatoid arthritis and ankylosing spondylitis patients

**DOI:** 10.1186/s13075-018-1598-8

**Published:** 2018-05-29

**Authors:** Katie L. Druce, Laraine Aikman, Maria Dilleen, Annie Burden, Piotr Szczypa, Neil Basu

**Affiliations:** 1 Arthritis Research UK Centre for Epidemiology, Faculty of Biology Medicine and Health, the University of Manchester, Manchester, M13 9PT UK; 2 Medical Affairs, Pfizer, Sandwich, UK; 3 Statistics, Global Product Development, Pfizer, Sandwich, UK; 4 Quanticate, Hitchin, UK; 5 Epidemiology Group, School of Medicine, Medical Sciences and Nutrition, University of Aberdeen, Aberdeen, UK; 6 Aberdeen Centre for Arthritis and Musculoskeletal Health, University of Aberdeen, Aberdeen, UK; 7 Arthritis Research UK/MRC Centre for Musculoskeletal Health and Work, University of Aberdeen, Aberdeen, UK

**Keywords:** Fatigue, Work disability, Rheumatoid arthritis, Spondyloarthritis

## Abstract

**Background:**

Work disability remains a significant problem in ankylosing spondylitis (AS) and rheumatoid arthritis (RA), despite biological therapy. This study aimed to test the hypothesis that the prevalent symptom of fatigue longitudinally predicts work disability among RA and AS patients commencing etanercept.

**Methods:**

Two observational studies, comprising RA and AS etanercept commencers, respectively, were analysed. Both provided data on work disability over 1 year and a comprehensive set of putative predictors, including fatigue. A series of repeated measures models were conducted, including baseline variables, visit (6/12 months), and the interaction between visit and each of the explanatory variables.

**Results:**

A total of 1003 AS and 1747 RA patients were assessed. For AS, fatigue was significantly associated with presenteeism (linear mixed model coefficient 3.75, 95% confidence interval (CI) 2.14 to 5.36) and activity impairment (2.62, 1.26 to 3.98), but not with work productivity loss (1.81, −0.40 to 4.02) or absenteeism (generalised linear mixed model odds ratio (OR) 1.18, 95% CI 0.92 to 1.51). In RA, fatigue was associated with presenteeism (coefficient 3.44, 95% CI 2.17 to 4.70), activity impairment (1.52, 0.79 to 2.26), work productivity loss (4.16, 2.47 to 5.85), and absenteeism (OR 1.23, 95% CI 1.02 to 1.49). The lack of significant interactions between fatigue and visit supported a consistent effect of baseline fatigue over time.

**Conclusions:**

Among patients beginning etanercept therapy, fatigue has a significant and independent effect on absenteeism, presenteeism, productivity loss, and activity impairment for RA patients and a significant but dimension-selective effect on work disability among AS patients.

**Trial registration:**

ClinicalTrials.gov, NCT00544557. Registered on 16 October 2007. ClinicalTrials.gov, NCT00488475. Registered on 20 June 2006.

## Background

Inflammatory rheumatic diseases, such as rheumatoid arthritis (RA) and ankylosing spondylitis (AS), are a substantial cause of work disability [1], a key outcome for both the patient and society. Estimates indicate that within 10 years of symptom onset up to 70% of patients become work disabled [2], rising to approximately 90% as disease duration increases [3].

Biological therapies have significantly minimised the physical sequelae of poorly controlled inflammation, such as joint destruction [4–6]. This in turn has been associated with an improvement in patient outcomes including those relating to work capacities [4–8]. Despite these gains, work disability remains an issue for the majority of RA and AS patients and it has become increasingly clear that the determinants of work disability in inflammatory diseases are not necessarily inflammatory in origin [9–11].

Fatigue, a patient priority inconsistently associated with inflammation [12], is possibly one such determinant. This symptom represents a persistent, unmanageable, and unpredictable issue [13–15] which is reported by up to 80% of patients with AS and RA. The impact of fatigue is multi-dimensional. For example, levels of physical activity are reduced and relationships are harmed [13, 14]. Yet the symptom is poorly managed, and many patients feel this subject is ignored by their clinicians. Subsequent attempts to self-manage by trial and error are typically unsuccessful [14, 16].

Anecdotally, patients have identified fatigue as a principal barrier to employment and productivity [13, 16]. The subsequent need to reduce work hours, relinquish roles, or even retire from work altogether have been attributed to the emotional, physical, and cognitive consequences of the symptom [3, 13, 16, 17]. However, the empirical evidence supporting these attributions is limited.

To date, analyses of the relationship between fatigue and work disability have generally examined cross-sectional data in small sample sizes [3, 18] and knowledge regarding the long-term nature of the relationship is sparse. Indeed, a previous systematic review of work disability predictors in RA surprisingly failed to identify any studies that have evaluated fatigue as a putative factor [19]. Furthermore, no study designs examining the impact of fatigue have employed a comprehensive measure of work disability which is necessary to adequately capture this complex multi-dimensional construct. To evaluate the different components, which include presenteeism (reduced performance at work due to ill health) and productivity in addition to the traditionally applied measure of absenteeism, suitably designed and detailed tools should be applied [20]. Finally, to our knowledge, no investigation has compared the fatigue-work disability relationship between distinct rheumatic disorders.

We have previously observed that patients commonly report continuing high levels of fatigue despite successfully attaining clinical remission (Disease Activity Score (DAS28) < 2.6) 6 months after commencing biologic therapy [21]. We now hypothesise that, in this current era of biological therapies, fatigue is independently associated with continuing levels of work disability among patients with inflammatory rheumatic diseases. Specifically, this study aimed to examine whether fatigue independently predicts different work disability dimensions, adjusting for clinical and psychosocial factors, over 1 year in working patient populations commencing etanercept, a commonly prescribed biologic. Furthermore, we obtained data from two near-identical studies on AS and RA to compare the relative impact of fatigue on work disability between these two common, but pathologically distinct, inflammatory rheumatic diseases.

## Methods

Data were obtained from 6518 patients recruited to one of two observational studies on patients with clinician-confirmed AS (*n* = 1663; clinical study number NCT00544557) or RA (*n* = 4855; clinical study number NCT00488475) who were commencing etanercept as part of routine clinical practice in Germany. Ethical approval for the original studies was obtained from the Ethics Committee of the State Chamber of Physicians (Landesaertzekammer; NCT00544557, 21 September 2007; NCT00488475, 15 September 2006).

Both cohorts were followed-up for 1 year. Data collected at baseline, 6 months, and 1 year contributed to this study. Of the variables captured, data obtained for these analyses comprised fatigue, work disability, demographics, clinical, and psychosocial status.

### Fatigue

Fatigue was measured using visual analogue scales (VAS) anchored from ‘no fatigue’ to ‘worst possible fatigue’. Although limited to providing a global assessment of fatigue, VAS have been extensively used and validated across most of the rheumatic disease spectrum. In the AS cohort, the VAS was extracted from the self-reported Bath Ankylosing Spondylitis Disease Activity Index (BASDAI) questionnaire and was thus scored 0–10 cm. In the RA cohort, the VAS was originally scored 0–100 mm but was converted into a 0–10 cm scale to facilitate comparability.

### Work disability

Work disability was measured using the six-item self-report Work Productivity and Activity Impairment—Special Health Problems (WPAI:SHP). The WPAI:SHP assesses the impact of the participant’s condition on work and daily activities for the past 7 days. The WPAI:SHP has been validated for use in both AS [22] and RA [23], and can be modified for any health problem by specifying the disease/condition of interest in the questions.

The questionnaire comprises four subscales: absenteeism, presenteeism, overall work impairment (absenteeism plus presenteeism), and daily activity impairment. Scores are transformed into impairment percentages ranging from 0 to 100%, with high scores indicating greater impairment and reduced productivity.

The absenteeism subscale was heavily skewed, with over 80% of patients reporting no absenteeism. This variable was therefore dichotomised (as 0 or > 0) for the analysis; all other subscales were treated as continuous variables.

### Demographics

Of the information captured at baseline, participant age, sex, and current working status (currently employed or not) were of interest.

### Clinical status

Physicians assessed disease activity using an 11-point Likert scale, from 0 ‘no disease activity’ to 10 ‘high disease activity’ in the AS cohort, and a 0–100 mm VAS from 0 mm ‘no disease activity’ to 100 mm ‘maximum possible disease activity’ in the RA cohort. C-reactive protein (CRP) was measured from blood samples taken at baseline.

### Psychosocial status

Self-reported pain and anxiety/depression were considered potential confounders. Pain was recorded by an 11-point Likert scale, from 0 ‘least possible pain’ to 10 ‘worst possible pain’ in the AS cohort, and by a 0–100 mm VAS from 0 mm ‘minimum possible pain’ to 100 mm ‘maximum possible pain’ in the RA cohort. Anxiety/depression was recorded using the anxiety and depression field of the European Quality of Life 5 Dimensions (EQ-5D) questionnaire. The EQ-5D has been validated for use in RA and AS populations [24, 25] and has three possible ordinal outcomes: no problems, some problems, and extreme problems.

### Analysis

In those participants who were employed at baseline (irrespective of proportion of work impairment due to absenteeism), repeated measures models (repeated by visit within subject) using a compound symmetry covariance structure were used to investigate the longitudinal relationship between each work disability score at 6 and/or 12 months and baseline fatigue. For absenteeism (dichotomised as 0 or > 0), a generalised linear mixed model was used with binomial distribution and logit link, while a linear mixed model was used for the remaining subscales.

Initial models included baseline fatigue and other potential baseline predictors as explanatory variables, visit (6/12 months), and the interaction between visit and each of the explanatory variables. The model was then manually stepwise reduced, removing the most non-significant interactions at each step and then the most non-significant main effects (unless there was a significant interaction with visit, in which case the main effect was retained to allow the total effect at each visit to be quantified). A nominal 5% level of significance was used, but terms were retained at the 10% level if they modified the fatigue effect.

All continuous variables included in the models were standardised to aid non-statistical comparison between the diseases. Such that the resultant regression coefficients should be interpreted as the number of standard deviations the dependent variable would change per one standard deviation increase in the predictor.

All analyses were carried out using SAS^©^ version 9.3.

## Results

Of 1663 AS patients, 1003 (60%) were working at baseline and could be included in this analysis. The majority (63%, *n* = 635) were male, with a mean age of 40.7 years (standard deviation 10.6). Of 4855 RA patients, 1747 (36%) were working at baseline and were eligible for this analysis. Just under three-quarters (72%) were female, with a mean age of 47.3 years (9.9) (Table 1).

**Table 1:** Baseline characteristics of the 1003 AS patients and 1747 RA patients who were working at baseline

Parameter		AS	*n*	RA	*n*
Age (years), mean (SD)		40.7 (10.6)	1003	47.3 (9.9)	1747
Female, %		36.7	1003	72.4	1747
Disease duration (years), mean (SD)		7.3 (7.8)	979	7.9 (7.6)	1654
Disease activity	0–10 Likert, mean (SD)	6.3 (1.7)	1000	–	
	0–100 mm VAS, mean (SD)	–		59.1 (18.0)	1724
CRP (mg/dL), median (min, max)		1.0 (0.0, 42.7)	906	0.9 (0.0, 20.0)	1534
Fatigue	0–10 cm VAS, mean (SD)	5.6 (2.4)	997	–	
	0–100 mm VAS, mean (SD)	–		57.4 (24.9)	1735
Pain	0–10 Likert, mean (SD)	6.3 (2.2)	990	–	
	0–100 mm VAS, mean (SD)	–		61.6 (21.4)	1736
Anxiety/depression (EQ-5D)	No problems, %	53.6	998	52.1	1738
	Some problems, %	42.7		44.4	
	Extreme problems, %	3.7		3.6	
WPAI:SHP					
% work time missed due to AS/RA^a^	No timed missed	61.9	776	61.3	1293
	Some time missed	38.1		38.7	
% impairment while working due to AS/RA^b^, mean (SD)		48.3 (26.9)	889	49.5 (27.1)	1530
% overall work impairment due to AS/RA^c^, mean (SD)		52.9 (29.8)	716	54.1 (29.0)	1171
% activity impairment due to AS/RA^d^, mean (SD)		53.4 (24.6)	990	54.0 (24.9)	1718

^a^Absenteeism

^b^Presenteeism

^c^Work productivity loss

^d^Activity impairment

AS, ankylosing spondylitis; CRP, C-reactive protein; EQ-5D, European Quality of Life 5 Dimensions; RA, rheumatoid arthritis; SD, standard deviation; VAS, visual analogue scale; WPAI:SHP, Work Productivity and Activity Impairment—Special Health Problems

Apart from the expected greater proportion of male AS versus RA patients, the cohorts were comparable at baseline. No relevant differences in clinical, psychosocial, or work disability domains were observed.

### Fatigue as a predictor of work and activity impairment in AS

Of the variables entered into the model, only baseline absenteeism (odds ratio (OR) 4.59, 95% confidence interval (CI) 2.91 to 7.25) and having some, versus no, problems with anxiety or depression (OR 1.77, 95% CI 1.12 to 2.80) were associated with a statistically significant increase in the odds of absenteeism at 6 and 12 months (Table 2). Although fatigue at baseline was associated with a small increase in the odds of absenteeism, the result was not significant (OR 1.18, 95% CI 0.92 to 1.51).

**Table 2:** Baseline variables predictive of WPAI:SHP subscales at 6 and 12 months in 1003 AS patients

Predictor		Odds ratio (95% CI)
Outcome: absenteeism		
Fatigue		1.18 (0.92 to 1.51)
% work time missed due to AS (> 0 vs 0)		4.59 (2.91 to 7.25)
Anxiety/depression	Some vs none	1.77 (1.12 to 2.80)
	Extreme vs none	0.87 (0.26 to 2.86)
	Extreme vs some	0.49 (0.15 to 1.57)
Visit 12 months vs 6 months		1.001 (0.70 to 1.44)
Predictor		Standardized beta-coefficient (β) (95% CI)
Outcome: presenteeism		
Fatigue		3.75 (2.14 to 5.36)
Male vs female		−4.65 (−7.62 to −1.68)
Age (years)		4.48 (3.07 to 5.89)
CRP		−1.83 (−3.53 to −0.13)
% impairment while working due to AS		6.25 (4.67 to 7.82)
Visit 12 months vs 6 months		−0.99 (−2.57 to 0.60)
Outcome: Work productivity loss		
Fatigue		1.81 (−0.40 to 4.02)
Male vs female		−3.57 (−7.29 to 0.15)
Age (years)		5.22 (3.52 to 6.93)
Pain		2.77 (0.34 to 5.20)
Pain at 12 months vs pain at 6 months		−2.49 (−4.49 to −0.49)
% overall work impairment due to AS		6.62 (4.53 to 8.71)
Anxiety/depression	Some vs none	3.76 (0.09 to 7.42)
	Extreme vs none	12.32 (1.38 to 23.26)
Visit 12 months vs 6 months		−1.42 (−3.42 to 0.59)
Outcome: Activity impairment		
Fatigue		2.62 (1.26 to 3.98)
Age (years)		5.07 (3.96 to 6.19)
CRP		−2.76 (−3.85 to −1.67)
% activity impairment due to AS		8.51 (7.14 to 9.87)
Anxiety/depression	Some vs none	3.27 (0.85 to 5.68)
	Extreme vs none	10.74 (5.35 to 16.14)
Visit 12 months vs 6 months		−1.10 (−2.35 to 0.15)

AS, ankylosing spondylitis; CI, confidence interval; CRP, C-reactive protein; RA, rheumatoid arthritis; VAS, visual analogue scale; WPAI:SHP, Work Productivity and Activity Impairment—Special Health Problems

Fatigue at baseline was a statistically significant predictor of presenteeism at 6 and 12 months (standardized beta co-efficient (β) 3.75, 95% CI 2.14 to 5.36) unit increase in impairment. Age (β 4.48, 95% CI 3.07 to 5.89) and presenteeism at baseline (β:6.25, 95% CI 4.67 to 7.82) were also associated with a statistically significant increase in presenteeism. Being male was associated with reduced impairment of presenteeism over time (β −4.65, 95% CI −7.62 to −1.68) as was baseline CRP level (β −1.83, 95% CI −3.53 to −0.13).

Increased work productivity loss over time was predicted by age (β 5.22, 95% CI 3.52 to 6.93) and work productivity loss at baseline (β 6.62, 95% CI 4.53 to 8.71), as well as having some (β 3.76, 95% CI 0.09 to 7.42) or extreme (β 12.32, 95% CI 1.38 to 23.26) problems with anxiety or depression, compared with no problems. Fatigue at baseline was not associated with work productivity loss (β 1.81, 95% CI −0.40 to 4.02) and, although pain was at 6 months (β 2.77, 95% CI 0.34 to 5.20), the significant interaction between visit and pain score at baseline suggests a negligible effect of baseline pain by 12 months.

Finally, increased activity impairment during the course of the study was associated with age (β 5.07, 95% CI 3.96 to 6.19), having some (β 3.27, 95% CI 0.85 to 5.68) or extreme (β 10.74, 95% CI 5.35 to 16.14) problems with anxiety or depression compared with no problems, having increased fatigue at baseline (β 2.62, 95% CI 1.26 to 3.98), and higher impairment at baseline (β 8.51, 95% CI 7.14 to 9.87). Higher baseline inflammation, as measured by CRP, was associated with a reduction in activity impairment at 6 and 12 months (β −2.76, 95% CI −3.85 to −1.67).

### Fatigue as a predictor of work and activity impairment in RA

In patients with RA, the likelihood of absenteeism at 6 and 12 months was significantly higher in those with more fatigue at baseline (OR 1.23, 95% CI 1.02 to 1.49), who were older (OR 1.20, 1.00 to 1.44), and who reported absenteeism at baseline (OR 4.75, 3.35 to 6.75). Greater inflammation at baseline was associated with a decreased likelihood of reporting absenteeism over the course of the study (OR 0.82, 95% CI 0.68 to 0.98) (Table 3).

**Table 3:** Baseline variables predictive of WPAI:SHP subscales at 6 and 12 months in 1747 RA patients

Predictor		Odds ratio (95% CI)
Outcome: absenteeism		
Fatigue		1.23 (1.02 to 1.49)
Age (years)		1.20 (1.00 to 1.44)
CRP		0.82 (0.68 to 0.98)
% work time missed due to RA (> 0 vs 0)		4.75 (3.35 to 6.75)
Visit 12 months vs 6 months		1.10 (0.83 to 1.46)
Predictor		Standardized beta-coefficient (β) (95% CI)
Outcome: presenteeism		
Fatigue		3.44 (2.17 to 4.71)
Age (years)		1.44 (0.26 to 2.63)
% impairment while working due to RA		9.36 (7.96 to 10.76)
% impairment at 12 months vs % impairment at 6 months		−1.98 (−3.39 to −0.58)
Visit 12 months vs 6 months		−1.16 (−2.57 to 0.24)
Outcome: Work productivity loss		
Fatigue		4.16 (2.47 to 5.85)
% overall work impairment due to RA		9.43 (7.74 to 11.12)
Visit 12 months vs 6 months		−0.60 (−2.64 to 1.44)
Outcome: Activity impairment		
Fatigue		1.52 (0.79 to 2.26)
Male vs female		−1.83 (−3.33 to −0.33)
Age (years)		3.25 (2.61 to 3.90)
% activity impairment due to RA		9.41 (8.59 to 10.23)
% activity impairment at 12 months vs % activity impairment at 6 months		−1.92 (−2.67 to −1.18)
Anxiety/depression	Some vs none	2.80 (1.40 to 4.20)
	Extreme vs none	5.81 (2.82 to 8.79)
Visit 12 months vs 6 months		−2.36 (−3.10 to −1.61)

CI, confidence interval; CRP, C-reactive protein; RA, rheumatoid arthritis; WPAI:SHP, Work Productivity and Activity Impairment—Special Health Problems

Increased presenteeism over time was significantly predicted by baseline fatigue (β 3.44, 95% CI 2.17 to 4.71) and age (β 1.44, 95% CI 0.26 to 2.63). Presenteeism at baseline was associated with a statistically significant increase in presenteeism at 6 and 12 months (Table 3).

Of all the putative predictors entered into the models, only fatigue (β 4.16, 95% CI 2.47 to 5.85) and baseline work productivity loss (β 9.43, 95% CI 7.74 to 11.12) were associated with increased work productivity loss over time. Increased activity impairment at 6 and 12 months was significantly associated with fatigue (β 1.52, 95% CI 0.79 to 2.26), older age (β 3.25, 95% CI 2.61 to 3.90), and having some (β 2.80, 95% CI 1.40 to 4.20) or extreme (β 5.81, 95% CI 2.82 to 8.79) problems with anxiety or depression, compared with no problems. Baseline activity impairment was associated with greater impairment over time (β 9.41, 95% CI 8.59 to 10.23 at 6 months), but the significant interaction between baseline activity impairment and visit suggested a reduced effect was present by 12 months.

### Comparison of fatigue as a predictor of work disability between RA and AS

The most consistent predictive factor for both populations was the baseline level of the predicted work outcome. Otherwise, although overall fatigue was a commonly identified independently significant predictor of work disability in both RA and AS patients, its effect appears more prominent among RA patients in relation to other putative predictors while, in AS patients, the effect of anxiety/depression was relatively stronger. In contrast, other variables such as disease activity and pain did not appear to be important in these study samples.

When considering the associations between fatigue and the different work disability components, both similarities and differences existed across RA and AS patients. For both diseases, fatigue was associated with presenteeism (AS: β 3.75, 95% CI 2.14 to 5.36; RA: β 3.44, 95% CI 2.17 to 4.71) and activity impairment (AS: β 2.62, 95% CI 1.26 to 3.98; RA: β 1.52, 95% CI 0.79 to 2.26). However, fatigue was identified as a significant predictor of all four work disability components under investigation in our RA population, while fatigue was not identified as an independent predictor of absenteeism or work productivity loss among AS patients. In both studies, no interactions between fatigue and visit were significant, suggesting a consistent effect of fatigue over the following 12-month period.

## Discussion

In this large study, fatigue had a significant and independent effect on all work disability dimensions at 6 and 12 months for patients with RA commencing etanercept. For AS patients who commenced etanercept, fatigue was identified as having a significant effect on presenteeism and overall activity impairment only. Across both studies, the lack of significant interactions between fatigue and visit suggests a consistent effect of baseline fatigue over the following 12-month period.

This study adds to the growing body of evidence highlighting the great importance of this previously ignored symptom among patients with inflammatory rheumatic diseases, and particularly emphasizes the economic impact of fatigue for both the individual and society as a whole. The existence of this relationship in the context of expensive biological treatment serves to enhance the economic issues further—despite their prescription, work disability remains an issue. The data presented herein support the case for targeting fatigue in parallel (employing, for example, non-pharmacological approaches) with a view to optimizing work-related outcomes and so providing even greater societal returns to the considerable pharmacological investments that global health systems have made in recent years. Furthermore, the study uniquely indicates that fatigue is importantly associated with work disability across two disparate disease populations, although the apparent differential effect on work disability dimensions should encourage some consideration of disease-specific issues during the development of appropriate interventions.

The few previous studies to quantitatively examine the relationship between RA and AS fatigue and work disability have been mostly cross-sectional and have all reported a significant association between these two factors [3, 18, 26, 27]. Of the longitudinal assessments, Zirkzee and colleagues found fatigue to be only weakly associated (*p* = .131) with deteriorating work status at 1 year among a small, likely underpowered, sample (*n* = 69) of Dutch patients newly diagnosed with inflammatory arthritis [28]. Similarly, fatigue was identified as a significant univariable, but not multivariable, predictor of receiving a work disability pension in a Norwegian study (*n* = 159) that aimed long-term work disability (the follow-up time point was 7 years) [29]. However, for patients, the predictors of shorter-term work disability are likely to be more informative in the context of preventative intervention development. Such future therapies may rescue patients from more immediate personal financial concerns related to their employment.

Pre-existing deficiencies in work disability measures were consistently the strongest predictors of the same outcome at 1 year. While this is intuitive, more surprising was the absence of independent associations between disease activity or pain and work disability.

This observation may reflect the success of biological therapies in targeting these factors during the follow-up of these cohorts and subsequently attenuating any association. Although fatigue may also be a mediator of the relationship between pain or disease activity and work disability, it is also plausible that no strong relationship may actually exist between these variables. Certainly, a previous systematic review only reported inconsistent evidence for a relationship between these traditional clinical factors [19].

In keeping with a previous report [30], we have shown that levels of fatigue and work disability are comparable between RA and AS working age populations. However, in this study, fatigue appears to differentially impact the domains of work impairment in RA and AS. One possible explanation for this difference may be the (expected) higher prevalence of males within the AS cohort. Work disability has previously been found to be dependent on the work status of the spouse [9], i.e. those married to a working partner are more likely to reduce/stop their own work when compromised by disease. In many cultures, including in Germany, it remains common for males to adopt the traditional role as the principal economic provider for the family unit. Thus, the threshold for work disability in response to fatigue could be higher as a result of their greater financial responsibilities. Another explanation is that the impact of fatigue may be greater for females compared with males. In rheumatic disease, fatigue is consistently reported to be greater among females for reasons as yet undetermined [31]. Finally, the nature of fatigue (and subsequent impact) may be distinct between AS and RA resulting from separate pathways.

Certainly, in terms of developing preventative interventions, the differential impact of fatigue as well as the more prominent association between anxiety/depression and work disability for AS compared with RA patients supports the delivery of disease-specific rather than generic interventions to prevent work disability.

A number of potential limitations should be considered when interpreting these results. Firstly, participants were new commencers of biological therapies and so were not representative of all AS and RA patients. The majority of RA and AS patients do not receive biological therapies [32, 33]. Such patients are typically characterised by high levels of disease activity and past disease-modifying anti-rheumatic drug failure. Data from both the German and UK biologics registers, which also recruit disease controls, indicate that RA patients not receiving biologicals are demographically similar, but AS patients tend to be younger [34–36]. Although it is possible that these results may not be generalisable, the population frame (which enables the selection of those patients with significant work disability despite a trial of biological therapy) is ideal to help us elucidate those determinants of work disability that are not necessarily fully related to inflammation (such as fatigue). We also acknowledge that generalisability of our identified associations may be influenced by cultural differences. For example, attitudes towards work [37], resilience factors [38], and availability of social security [26] support may influence the levels of work disability and all may vary between countries (although this may not necessarily confound the relationship with fatigue).

Secondly, in the absence of a control group, we cannot quantify the effect of etanercept on either fatigue or work productivity. Nevertheless, additional analyses showed that, among those still taking etanercept at 12 months (RA *n* = 1091, AS *n* = 689), average reductions of 20% were seen in presenteeism, work productivity loss, and activity impairment in both cohorts while absenteeism reduced by an average of 8–15% in RA and AS, respectively (data not shown).

Furthermore, work impairment is likely multifactorial in origin. While we were able to access data on a number of factors which are commonly implicated with this outcome, a number of other potentially interesting variables, for example sleep disturbance, co-medications, and disease severity, were not captured by the study and could not be incorporated into this analysis. Thirdly, given the observational design of this study, causality cannot be inferred although it seems quite possible given what we already know from patient reports. Finally, the psychosocial measures available to us for this secondary analysis provided only non-specific global measures of the underlying constructs of interest. For example, the EQ-5D does not enable the relative contributions of anxiety and depression to be separately determined.

Similarly, we acknowledge that our use of a VAS to capture fatigue may not have fully captured this complex construct relative to multi-dimensional measures, such as the Bristol Rheumatoid Arthritis Fatigue Multi-Dimensional Questionnaire (BRAF MDQ) or Chalder Fatigue Questionnaire (CFQ) [39]. Fatigue VAS scales are, however, considered suitable for use for those seeking to obtain a global measurement of fatigue, and are sensitive to change and are commonly used, aiding comparison with other diseases [39].

Future studies should seek to validate these finding using different fatigue instruments in different sampling frames. The development of interventions to reduce work disability among patients with arthritis is a key priority for patients and researchers alike. The targets for such interventions are likely to be multiple, but this study provides evidence that fatigue-specific measures should be a key component in the development of any future interventions. Existing evidence supports the efficacy of non-pharmacological interventions such as exercise and cognitive behavioural therapies for fatigue in the context of inflammatory rheumatic diseases [40]. A better understanding of the biology of fatigue will be important for the development of alternative therapies in the future.

## Conclusions

This is the largest study to examine the longitudinal association between fatigue and work impairment and, by selecting two cohorts which underwent near-identical interventions and data collection procedures, the first to enable the comparison of this relationship between two different rheumatic diseases. We have demonstrated that, among patients beginning etanercept therapy, fatigue has a significant effect on the absenteeism, presenteeism, productivity loss, and activity impairment of patients with RA, and a significant but dimension-selective effect on work disability among AS patients. In addition to pharmacological treatment of inflammation, fatigue-alleviating interventions such as cognitive behavioural therapy and exercise should be tested in both RA and AS patients with a view to modifying the course of this crucial outcome.

## Abbreviations

AS: Ankylosing spondylitis
BASDAI: Bath Ankylosing Spondylitis Disease Activity Index
BRAF MDQ: Bristol Rheumatoid Arthritis Fatigue Multi-Dimensional Questionnaire
CFQ: Chalder Fatigue Questionnaire
CI: Confidence interval
CRP: C-reactive protein
DAS28: Disease Activity Score for 28 joints
EQ-5D: European Quality of Life 5 Dimensions
OR: Odds ratio
RA: Rheumatoid arthritis
VAS: Visual analogue scales
WPAI:SHP: Work Productivity and Activity Impairment—Special Health Problems
